# Real Time Monitoring of Temperature of a Micro Proton Exchange Membrane Fuel Cell

**DOI:** 10.3390/s90301423

**Published:** 2009-03-03

**Authors:** Chi-Yuan Lee, Shuo-Jen Lee, Yuh-Chung Hu, Wen-Pin Shih, Wei-Yuan Fan, Chih-Wei Chuang

**Affiliations:** 1 Department of Mechanical Engineering, Yuan Ze Fuel Cell Center, Yuan Ze University, Taoyuan, Taiwan, R.O.C. E-Mails: mesjl@saturn.yzu.edu.tw; s975040@mail.yzu.edu.tw; s937257@mail.yzu.edu.tw; 2 Department of Mechanical and Electro-Mechanical Engineering, National ILan University, ILan, Taiwan, R.O.C.; E-Mail: ychu@niu.edu.tw; 3 Department of Mechanical Engineering, National Taiwan University, Taipei, Taiwan, R.O.C.; E-Mail: wpshih@ntu.edu.tw

**Keywords:** Si-MHA, MEMS, micro fuel cell, GDL, RTD sensor

## Abstract

Silicon micro-hole arrays (Si-MHA) were fabricated as a gas diffusion layer (GDL) in a micro fuel cell using the micro-electro-mechanical-systems (MEMS) fabrication technique. The resistance temperature detector (RTD) sensor was integrated with the GDL on a bipolar plate to measure the temperature inside the fuel cell. Experimental results demonstrate that temperature was generally linearly related to resistance and that accuracy and sensitivity were within 0.5 °C and 1.68×10^−3^/°C, respectively. The best experimental performance was 9.37 mW/cm^2^ at an H_2_/O_2_ dry gas flow rate of 30/30 SCCM. Fuel cell temperature during operation was 27 °C, as measured using thermocouples in contact with the backside of the electrode. Fuel cell operating temperature measured *in situ* was 30.5 °C.

## 1. Introduction

Fuel cells have been increasingly miniaturized and are common in portable electronic products, including cellular phones and PDAs. Silicon-based substrates are highly compatible with micro-electro-mechanical-systems (MEMS) technology [1–2]. Porous silicon has been utilized as the gas diffusion layer (GDL) in fuel cells, replacing traditional carbon cloth or carbon paper [3, 4, 5]. Porous silicon has also been used to produce proton exchange membranes [6].

Electrochemical etching with hydrofluoric acid has been studied since 1956. In 1990, Lehmann [7] characterized porous silicon in detail, and, in 1996, Lehmann [8] investigated the development of a porous silicon array structure, indicating that etching depends on electrolyte concentration, electrolyte temperature, silicon doping density and current density. Such structures are classified into three regimes according to the mean dimensions of the porous silicon. The mean dimension of the microporous regime is <2 nm; that of the mesoporous regime is 2–50 nm, and that of the macroporous regime is >50 nm. Kleimann [9] produced a macroporous array that was 42 μm wide and 200 μm deep. According to Kleimann’s findings, porous silicon etching can be utilized to generate a structure with a high aspect ratio at a lower cost than that associated with deep reactive ion etching (DRIE).

Numerous studies have measured important factors concerning the effects of cell temperature, fuel temperature, and fuel humidity, as well as other factors associated with cell performance [10, 11, 12, 13]. In this work, a resistance temperature detector (RTD) sensor was integrated into the GDL on a bipolar plate to measure the temperature inside a micro fuel cell.

## 2. Methodology

Wet etching was applied to produce fuel channels in a micro fuel cell. Dry etching was then used to generate silicon micro-hole arrays (Si-MHA). In this investigation, hole size and depth were controlled. After the Si-MHA were formed, platinum (Pt) was deposited on the surface holes as a catalyst of the fuel cell increasing the conductivity of the silicon. Part of the Pt metal layer was formed as a micro thermal sensor.

### 2.1. Theory of Thermal Sensors and Characteristics of Platinum

As a soft and silvery-white metal, Pt is extremely malleable [14], and has a resistance that varies linearly over a large temperature range of −260–1,000 °C. Even when the ambient temperature exceeds 1,000 °C, it remains stable and does not undergo significant physical or chemical changes. The error range is at minimum ±0.06 % (or ±0.15 °C) at 0 °C. Notably, Pt cannot be etched in strong acid or alkali, with the exception of *aqua regia*. Therefore, Pt is the material of choice for thermal sensors.

The resistance of a general metal is expressed as:

(1) $$R=\rho \frac{L}{A}$$

where *R* is resistance (Ω), *ρ* is resistivity (Ω-m), *L* is wire length (m), and *A* is wire cross-sectional area (m^2^). The resistivity of Pt is 1.042 × 10^−9^ Ω-m at room temperature. When used in a micro thermal sensor, the temperature coefficient of resistance of Pt varies with thin film thickness and ranges between 0.00375–0.00385. If the temperature variation range of the RTD is linear, then the relationship between measured resistance and temperature change is given by:

(2) $$R_{t}=R_{i}(1+\alpha_{T}\;\;\Delta T)$$

Equation (2) can be rewritten as:

(3) $$\alpha_{T}=\frac{R_{t}-R_{i}}{R_{i}(\Delta T)}$$

where *R_t_* is resistance at *t* °C, *R_i_* is resistance at *i* °C, and *α_T_* is sensitivity (1/°C) [15, 16].

### 2.2. Flow Field Design

**Figure 1.**
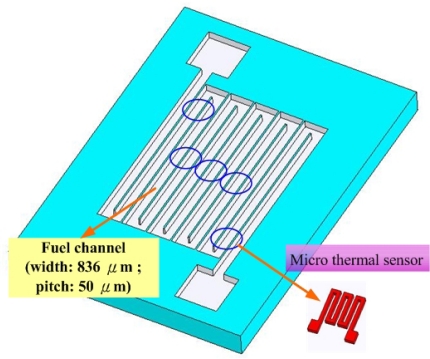
Form of flow channel with micro thermal sensor.

**Figure 2.**
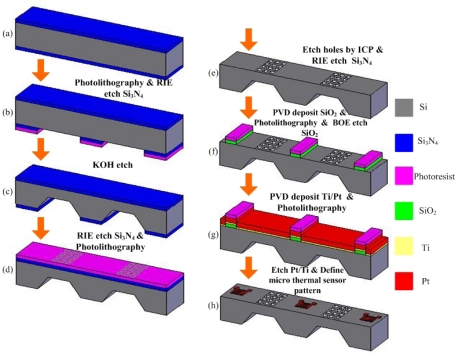
Fabrication flowchart.

Hoogers [17] demonstrated that the performance of a serpentine flow field on a fuel cell was better than other flow field configurations (meshed and interdigitated) in some cases, because the fuel (gas or liquid) was driven strongly to flow around the active area of the fuel cell. Hence, a serpentine flow field was applied in the design in this study, as displayed in Figure 1. An N-type thickness of 525±25 μm, and a (100)-oriented double-side polished wafer was used. After the low pressure chemical vapor deposition (LPCVD) oxidation of Si_3_N_4_ on the silicon wafer (5,000 Å thick), one side of the silicon was processed photolithographically. Reactive ion etching (RIE) was then utilized to transfer the pattern in Figure 1, as in the wet KOH etching process. This process was employed to etch a 450 μm-thick silicon layer. The remaining thickness of silicon constitutes the GDL, with a thickness of 50–70 μm and width of 200 μm. Figures 2a∼c depict the details of the process.

### 2.3. Standard Deviation of the Experiment

In this experiment, standard deviation for temperature and resistance are given by:

(4) $$S=\sqrt{\frac{1}{n-1}\sum {\left(x_{i}-\bar{x}\right)}^{2}}$$

where *S* is standard deviation, *x_i_* are particular values, *x̄* is the mean of all values, and *n* is sample size (number of values) [18].

## 3. Fabrication

Techniques described elsewhere [7, 19] were adopted to design square holes of side 10 μm, and form fuel channels with vertical walls. Etching time and current density were important parameters. In the proposed design, square holes of size 10 μm were fabricated by DRIE. Figure 2 depicts the Si-MHA fabrication process flow. The flow field process was performed as shown in Figures 2a∼c, the other side of the wafer was patterned lithographically, 10 μm square at a pitch of 15 μm covered the defined area, over which a 200 μm-thick GDL was formed (Figure 2d), and then was transferred in Si_3_N_4_. The KOH wet etching process on (100)-oriented silicon was anisotropic. The Si_3_N_4_ was removed from the fuel field side of the wafer by RIE after the fuel channel was formed. To ensure that the Si-MHA goes through to the flow field, DRIE was applied to reach the purpose have through-holes. Figure 2e displays the Si-MHA through to the flow field. A circle 10 μm in diameter was patterned lithographically on top of each flow channel. The pattern (200 μm×13140 μm) was transferred using Si_3_N_4_.

**Figure 3.**
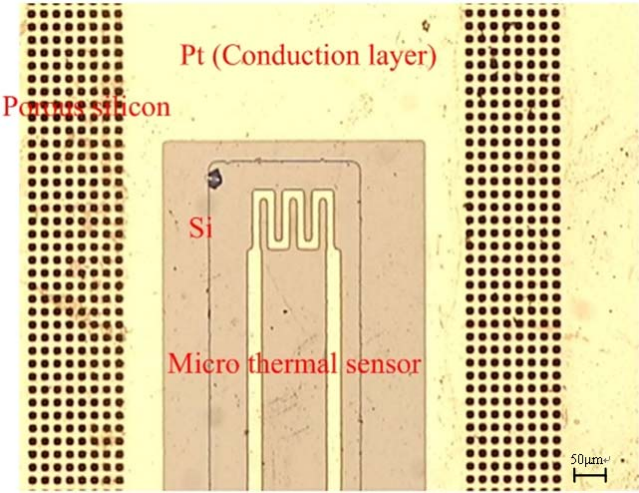
Optical microscopic photograph of the micro thermal sensor combined with the Si-MHA.

After the Si-MHA were produced, the wafer was metallized on the Si-MHA by depositing a layer of Ti/Pt (20 nm/70 nm). The Pt acted as the current collector and micro thermal sensor. Physical vapor deposition (PVD) was employed to deposit the Pt and wet etching was used to produce a conductive layer and micro thermal sensor. A photoresist was adopted as the etching mask, ensuring that the Pt remained on the surface of the Si-MHA. An etching mask was utilized in the lithography process with an exposure process. Figures 2e∼h present the process flow in detail. The micro thermal sensor and Si-MHA were fabricated, as shown in Figure 3.

## 4. Experimental

**Figure 4.**
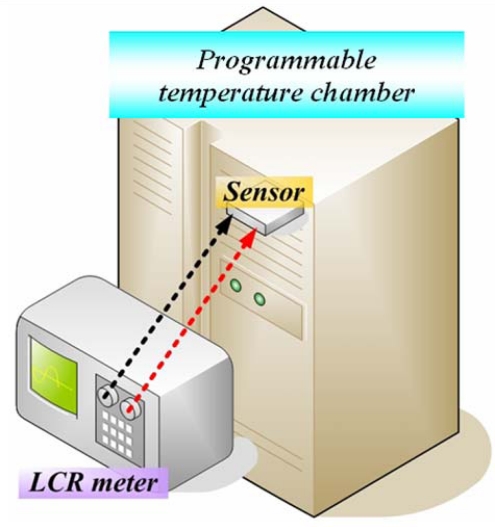
Experimental setup for calibrating the micro thermal sensor.

**Figure 5.**
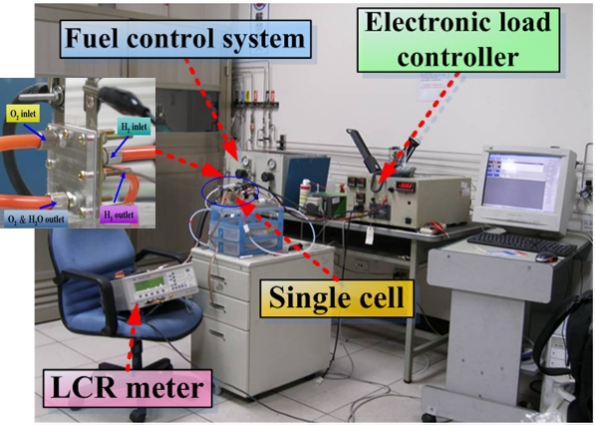
Experimental setup for measuring fuel cell performance.

In this experiment, the temperature of micro thermal sensor was measured and ranged from 25–45 °C. Hydrogen flowed on the anode side and oxygen flowed on the cathode side. Hydrogen and oxygen flows rates were 10 and 30 SCCM. The humidify parameter was increased from 20 °C to 40 °C. The active area of the fuel cell was 1.82 cm^2^ (1.3 cm×1.4 cm). The proton exchange membrane was obtained from Ion Power Co. The Pt loading was 0.5 mg/cm^2^. Figure 4 presents the experimental setup for calibrating the micro thermal sensor, and measuring resistance using a 4230 LCR meter. The frequency of the LCR meter was 1 kHz; the meter used a 4-terminal probe connection. In fuel cell performance tests, the fuel cells were connected to the fuel control system; the electronic load controlled the fuel feed rate, and the experimental setup for measuring fuel cell performance is shown in Figure 5.

## 5. Results and Discussion

### 5.1. In-situ measurement of temperature

**Figure 6.**
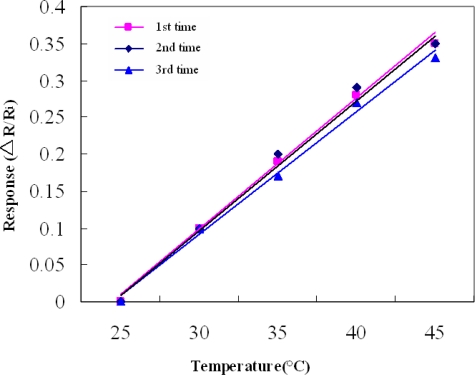
Normalized temperature response of the micro sensor.

Experimental results indicate that temperature was almost linearly related to resistance. Accuracy and sensitivity of the micro thermal sensor were 0.5 °C and 1.68×10^−3^/°C, respectively. Micro thermal sensor accuracy was defined based on temperature in the accuracy range (0.5 °C) of the programmable temperature chamber. Figure 6 shows the normalized temperature response of the micro thermal sensor, and the sensor was measured three times, and these response curves were very coincidental and had high reproducibility; standard deviation was 0.037212. Experimental data reveal that temperature was almost linearly related to resistance. Fuel cell temperature during operation was 27 °C, as measured using thermocouple in contact with the electrode backside. Temperature measured in situ during fuel cell operation was 30.5 °C.

### 5.2. Fuel cell performance

Performances of the fuel cell with 10 μm holes were compared under the following operating conditions: (a) at 15 °C (with no humidity) and 20 °C, 30 °C, 40 °C (with humidity); (b) hydrogen and oxygen flows rates at both 10 and 30 SCCM; and (c) without and with a micro thermal sensor at 15 °C (no humidity) and a flow rate of 30 SCCM.

**Figure 7.**
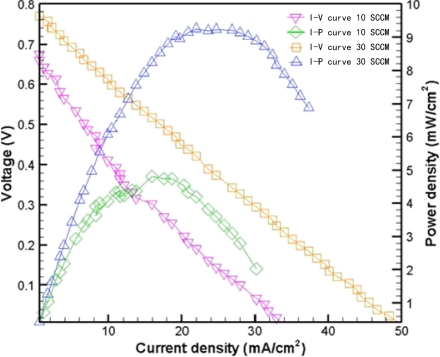
Experimental results with 10 μm holes at 15 °C (no humidity) and flow rates of 10 SCCM and 30 SCCM.

**Figure 8.**
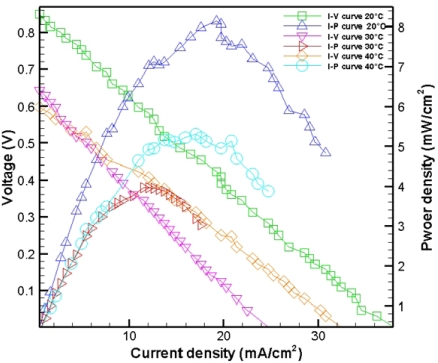
Experimental results obtained with 10 μm holes at 20 °C, 30 °C, 40 °C (humidity) and a flow rate of 30 SCCM.

**Figure 9.**
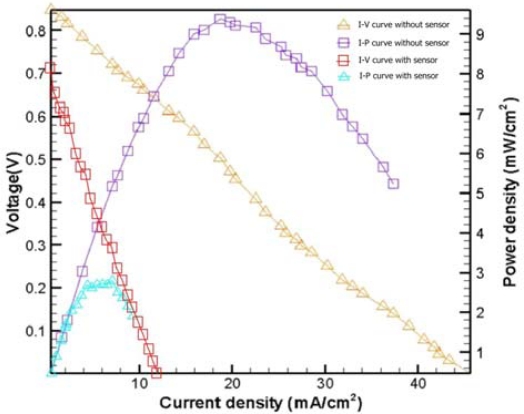
Performance results with 10 μm holes in the fuel cell without and with a micro thermal sensor at 15 °C (no humidity) and flow rate of 30 SCCM.

Figure 7 presents experimental results with 10 μm holes at 15 °C (no humidity) and flow rates of 10 SCCM and 30 SCCM. The maximum power density was approximately 9.25 mW/cm^2^, with a flow rate of 30 SCCM. Figure 8 shows experimental results obtained with 10 μm holes at 20 °C, 30 °C, 40 °C (humidity) and a flow rate of 30 SCCM. The maximum power density was approximately 8.13 mW/cm^2^ at 20°C. Figure 9 compares the performance obtained without a micro thermal sensor with that obtained with a micro thermal sensor on the anode electrode at 15 °C (no humidity) and a flow rate of 30 SCCM. The maximum power density of the fuel cell without and with a micro thermal sensor on the anode electrode was 9.37 mW/cm^2^ and 2.15 mW/cm^2^, respectively. Notably, the local temperature could be measured because a micro thermal sensor has an insulating layer on both sides, explaining why the insulated area likely decreased the reaction area and fuel cell performance. Table 1 summarizes the experimental results for Figure 7 to 9.

## 6. Conclusions

Si-MHA were fabricated on a defined area of the flow field. The silicon wafer combined the flow field and GDL. The silicon holes had 10 μm diameters and were fabricated under various operating conditions. The micro thermal sensor formed as the catalyst was deposited. Furthermore, the performance of a working fuel cell and its internal temperature were measured. The best fuel cell performance was 9.37 mW/cm^2^ at 502 mV without micro thermal sensors on the anode electrode, at a flow rate of 30 SCCM at 15 °C (no humidify); the anode electrode was integrated with 10 μm of Si-MHA in the fuel field. In situ monitoring of temperature during fuel cell operation was 30.5 °C.

## Figures and Tables

**Table 1.** Experimental results obtained under various operating conditions.

	**At 15 °C (no humidity) with 30 SCCM**	**At 20 °C (humidity) with 30 SCCM**	**Without a micro thermal sensor at 15 °C (no humidity) with 30 SCCM**	**With a micro thermal sensor at 15 °C (no humidity) with 30 SCCM**
**Voltage**	421 mV	423 mV	502 mV	302 mV
**The maximum power density**	9.25 mW/cm^2^	8.13 mW/cm^2^	9.37 mW/cm^2^	2.15 mW/cm^2^
